# SimAlba: A Spatial Microsimulation Approach to the Analysis of Health Inequalities

**DOI:** 10.3389/fpubh.2016.00230

**Published:** 2016-10-21

**Authors:** Malcolm Campbell, Dimitris Ballas

**Affiliations:** ^1^GeoHealth Laboratory, Department of Geography, University of Canterbury, Christchurch, New Zealand; ^2^Department of Geography, University of Sheffield, Sheffield, UK; ^3^Department of Geography, University of the Aegean, Mytilene, Greece

**Keywords:** spatial microsimulation, urban health inequalities, health policy, Scotland, geographic information systems, small area microdata

## Abstract

This paper presents applied geographical research based on a spatial microsimulation model, *SimAlba*, aimed at estimating geographically sensitive health variables in Scotland. *SimAlba* has been developed in order to answer a variety of “what-if” policy questions pertaining to health policy in Scotland. Using the *SimAlba* model, it is possible to simulate the distributions of previously unknown variables at the small area level such as smoking, alcohol consumption, mental well-being, and obesity. The *SimAlba* microdataset has been created by combining Scottish Health Survey and Census data using a deterministic reweighting spatial microsimulation algorithm developed for this purpose. The paper presents *SimAlba* outputs for Scotland’s largest city, Glasgow, and examines the spatial distribution of the simulated variables for small geographical areas in Glasgow as well as the effects on individuals of different policy scenario outcomes. In simulating previously unknown spatial data, a wealth of new perspectives can be examined and explored. This paper explores a small set of those potential avenues of research and shows the power of spatial microsimulation modeling in an urban context.

## Introduction

*SimAlba* is a spatial microsimulation model, which has been used to estimate geographically sensitive health variables for Scotland’s largest city, Glasgow. Spatial microsimulation is now a well-established method in geography for public policy analysis in a wide range of domains (1, 2). Building on these efforts, *SimAlba*^1^ has been developed in order to answer a variety of “what-if” policy questions pertaining to health policy in Scotland. We aim to show how this data could be (and have been) used to create “what-if” policy scenarios. A “what-if” policy scenario is an estimation of what may happen to health outcomes as a result of a hypothetical change in policy using modeled data.

There is a significant body of literature describing the uses of complex statistical models to analyze social and spatial inequalities in a variety of contexts. Specifically, the use of spatial microsimulation models (3–8) provide a new perspective on existing data sources and contribute to the relevant academic literature as well as applied health policy analysis efforts offering an opportunity to estimate previously unknown data as well as to analyze both individuals and areas simultaneously.

**Figure 1:**
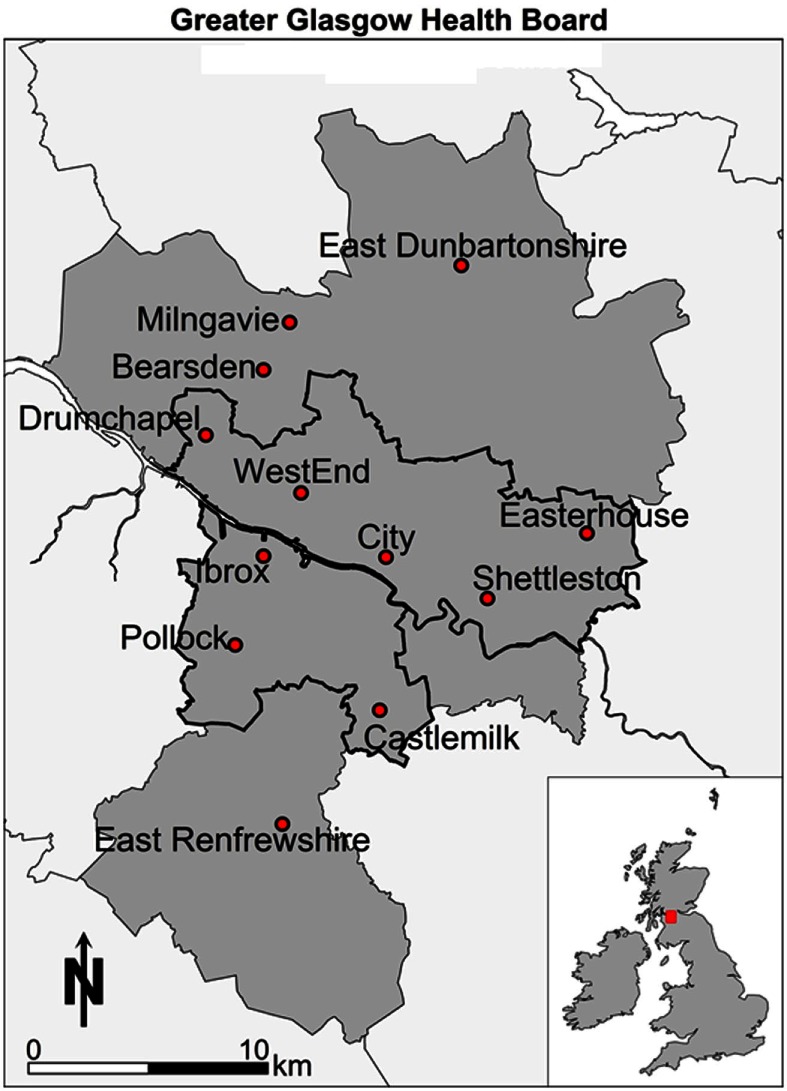
**Reference map**.

This paper aims to further demonstrate how spatial microsimulation can be used to estimate previously unavailable data and then to show how this data can be analyzed and visualized, using geographic information systems (GISs), to illuminate both the social and the spatial patterns in health-related behavior and outcomes in Glasgow, Scotland (see Figure 1). This paper forwards a new small area perspective on health-related variables in Scotland, showing how Scottish Health Survey (SHS) and Census data for Scotland can be combined to create a powerful policy modeling and visualization framework.

The paper is organized as follows: it begins by painting the health landscape of the study area; then giving an introduction to the microsimulation literature and explaining how spatial microsimulation can be operationalized in simple terms. Some outputs of the *SimAlba* model are then presented and explored, particularly focusing on the health-related variables created. A discussion of the relevance of the results simulated follows; concluding with directions for future research and the policy implications of the analysis presented.

## A Background to the Health Landscape in Scotland

The recent past has been marked by a series of deteriorations in Scottish health relative to the rest of Europe, which has led to Scotland being labeled as “the sick man of Europe.” This label has been applied to Scottish health more recently, signifying the noticeable divergence from the 1950s onward in terms of health compared with the rest of Europe. Glasgow, in particular, exhibited the highest levels of self-reported bad or very bad general health and psychological distress for both men and women compared across 32 other Europe metropolitan areas (9). The “Scottish Effect” (10) or the “Glasgow Effect” (11) details the excess mortality in Scotland and Glasgow, in particular, even after accounting for socioeconomic circumstances. This suggests that Scotland is peculiar in regards to population health, and that this effect may be even stronger in Glasgow; hence the focus in this paper on the urban area of this city. In other words, after taking account of deprivation, there is still an excess of mortality in Scotland compared to England and Wales (12). This issue is well-studied. For example, a report on Scottish health (13), identified “risk factors” in Scotland as tobacco, alcohol, low fruit and vegetable intake, physical activity levels, and obesity. More broadly, within the UK, there has long been ample evidence on the existence of health inequalities, especially, since the highly influential Black Report (14) that highlighted health inequalities by both place and socioeconomic status that continue to exist and persist over time (15) in the UK. Furthermore, when compared to the rest of Great Britain (GB) or the UK (16, 17) or its western European neighbors (18), Scotland does not do well. There have been many studies examining these broader country level differences over time between Scotland and the rest of GB [for a recent example comparing mortality patterns, see Ref. (12)].

Looking in more depth at Glasgow, the evidence of a specific “Glasgow Effect” as discussed above is a particular concern for this paper. A specific cause of concern is that premature mortality is 30% higher in Glasgow compared to similarly deprived UK cities (11). This paper adds to the understandings of why this may be the case by estimating previously unknown data. For example, discussion around the importance of alcohol consumption or drug use as contributing to half of the excess observed (19), with much of the deprivation potentially unmeasured, points to the usefulness of small area estimates to fill this gap. The specific spatial patterning of deprivation in Glasgow has been examined as a possible cause of the “Glasgow Effect”; evidence suggests that there is a strong impact of deprivation of surrounding areas on health outcomes (20) but not quite as originally hypothesized by McCartney et al. (21) as a concentrated monoculture. As McCartney et al. (21) explains, there are 17 possible explanations for the unique situation in Glasgow, concluding that understanding of the Scottish mortality patterning requires, as well as a clear focus on behaviors, an understanding of the most “upstream” determinants of health, to which spatial microsimulation can add some important value. Previous analysis of poverty and benefit take-up show that there are some geographical patterns, but only at unitary authority level (22), noting that the “worst” areas are concentrated around Glasgow combined with relative affluence nearby. Other work examining the geography of disadvantage in Glasgow (23) notes the persistence of disadvantage in areas in the east end (Shettleston, Easterhouse) as well as to the northwest (Drumchapel) and to the South (Castlemilk) and southwest of the center (Pollok) in the 1970s, 1980s, and 1990s. Of particular note is that Glasgow performs worse on all the deprivation-related variables compared to the Scottish average and the persistence of disadvantage, in particular, small areas of Glasgow. This pattern of higher deprivation in Glasgow continues, linking it with mortality rates, showing a strong bivariate relationship across Scotland; in other words, spatial proximity to deprivation is important for mortality outcomes (24). Qualitative evidence from Glasgow also points to the importance of area on health behaviors, that poorly resourced, stressful environments with strong community norms may foster smoking as well as undermining attempts to increase cessation rates (25). Moreover, the perceptions, as well as the health outcomes in neighborhoods in Glasgow have a social gradient, as outlined by Sooman and Macintyre (26), such that perceptions of an area can influence health outcomes. Overall, we can see the pattern of evidence pointing to the importance of area influence on health outcomes in Glasgow.

The role of smoking, alcohol consumption, diet, and physical activity in explaining socioeconomic differentials in mortality in the west of Scotland noted the importance of these behaviors for longer-term outcomes (27). Thus, having estimates of such behaviors at small area level can help increase understanding of the broader forces of health inequality associated with health behaviors. A Scottish specific issue is the role that alcohol plays in contributing to poor health outcomes linked to the minimum pricing of alcohol as a policy response (28). Scotland has among the highest alcohol-related deaths in Western Europe (29), although this has been falling since the 1990s. Scotland also embarked on a smoke-free policy, designed to reduce exposure to secondhand smoke. Evidence has shown that it has been a success (30) as well as having none of the hypothesizing negative outcome, such as more smoking in the home or economic impacts on businesses. Of particular relevance is the debate around the independence question for Scotland. Although the outcome was a “no,” there is still significant potential for further departure with respect to health policy compared to the rest of the UK (31).

Therefore, we can see that Glasgow has been the subject of much research into health inequalities as well as economic and social inequality. We add estimated health variables to this body of work at a small area level to further enhance knowledge and to highlight relevant social and spatial patterns and inequalities.

## A Brief Background to Spatial Microsimulation Modeling in Health

Spatial microsimulation is an established methodology in the social sciences with a long successful history in Economics since the late 1950s and with more recent significant developments in other disciplines, including geography in the last three decades (1, 2). In particular, there have been significant advances in spatial microsimulation models, in other words, adding geography to models (32). This adds to the potential uses of microsimulation, for example, by allowing assessment of area-based policies relating to social and health policy (3, 7, 33). Additionally, the geographic distribution of health-related variables can be simulated (3–6, 34), not just the socioeconomic or demographic patterns aspatially. This allows previously unknown small area spatial patterns to be investigated, and the spatial effects to be considered in concert with the socioeconomic and demographic factors. Building on these efforts, *SimAlba* has been developed in order to answer a variety of “what-if” policy questions pertaining to health policy in Scotland, with geography included as a key element. The *SimAlba* model has previously been used to estimate and model in the economic sphere (35, 36). We add to this literature by focusing on health.

## Data and Methods: SimAlba – A Spatial Microsimulation Model

The *SimAlba* model was developed with the use of data from the Census of Population 2001 and the SHS 2003. The Census of Population is carried out decennially, while the SHS 2003 was the third survey of Scottish health (after 1995 and 1998) and included all ages. Each SHS samples a new set of addresses and has both an adult and child component with a total of 8,148 adults and 3,324 children interviewed on a variety of health conditions and behaviors as well as socioeconomic and demographic information. The health variables include: smoking and alcohol consumption, physical activity, dental health, general health, and many others.

It is important to point out that the time periods of data collection (2001 and 2003) do not match precisely, but in the absence of any other temporally consistent health data, for Scotland, this is a pragmatic compromise. Spatial microsimulation uses the data contained in the SHS and “upscales” it to reflect the populations of census areas as closely as possible. This can be achieved using a process called deterministic reweighting (3, 8, 37). Deterministic reweighting has become an established method for estimating health variables in multiple contexts such as area smoking prevalence (4, 6) or obesity prevalence (38). Spatial microsimulation works by using a series of constraints that are used to construct the model, and which must be present in both datasets; this limits the potential constraint options available. A constraint variable is chosen by either using the literature or a more formal regression approach to see which variables in the datasets are most correlated with the variable to be predicted. Therefore, the choice of the constraints, though informed by the literature and other empirical research, must be pragmatic. Constraints are keys to the model set up (39) and, therefore, an important part of the spatial microsimulation modeling process.

*SimAlba* uses age, sex, marital status, illness, qualifications, economic activity, tenure, and an employment classification (National Socioeconomic Classification, NSSEC) as constraints. Note that the deterministic reweighting process is not explained in depth in this paper for reasons of brevity [for more details, see Ref. (36)]. The method is deterministic as it produces the same output for the same input data, which were an important consideration for policy end users. The stylized formula that can be applied to create microdata is NWi = Wi × CENij/SHSij.

The equation is constructed as follows: a new weight (NW) for individual i is calculated by multiplying the weight (W) for individual i by element ij of the Census table divided by element ij of the SHS table. This process is completed iteratively until a suitable level of convergence is reached, and NWi is the number of a particular individual created for a specific small area in Scotland. The process was followed to adjust the weights of individuals in the SHS to match census output areas (OAs) populations, which have a minimum population of around 40 households or 100 individuals. The end result is a spatially simulated dataset, which previously did not exist and which can now be used as the basis for further analysis.

Microsimulation has been used to estimate many different types of data in multiple contexts as discussed above. One of the key points of concern in the literature pertains to the reliability and accuracy of the microsimulated data. There is now a growing body of evidence showing that the technique provides robust estimates of health-related variables in particular (6, 38, 40). *SimAlba* has been internally and externally validated (see Figure 8) and has demonstrated that it provides robust data (35, 36). From Figure 8, it can be seen that the model produces estimates within 10% error, with most of the data falling close to the 45° line, signifying an exact match.

## Spatial Microsimulation Model Outputs: Estimating Health Behaviors and Outcomes

This section shows some of the microsimulated data tabulated and mapped so as to give a small snapshot of the type of data that can be produced by *SimAlba* and its policy relevance. Several of the variables simulated are now visualized using a quintile distribution, which can help us to better highlight the extremes of the spatially simulated data. Q1 refers to the highest values, Q5 the lowest in the distribution of variables. Only a small fraction of the data that can be mapped is, as any variable in the SHS can, potentially be simulated using the *SimAlba* algorithms.

In this paper, we demonstrate the relevance of the outputs of models like *SimAlba* to policy debates briefly discussed above by focusing on smoking prevalence, subjective well-being, alcohol consumption, and obesity. We therefore pose five policy relevant research questions that are readily applicable to spatially microsimulated data. Specifically, we demonstrate how models like *SimAlba* can be used to address research questions such as:

1. Which OAs in Glasgow have the greatest proportions of “unhappy” people?
2. Which areas have the greatest proportions of obese people?
3. Where do those men drinking over the daily limits reside?
4. What is the distribution of smokers in Greater Glasgow and to what extent is this altered by income?
5. Which OAs do those people who exhibit several simultaneous “unhealthy” characteristics reside in the greatest proportions?

**Figure 2:**
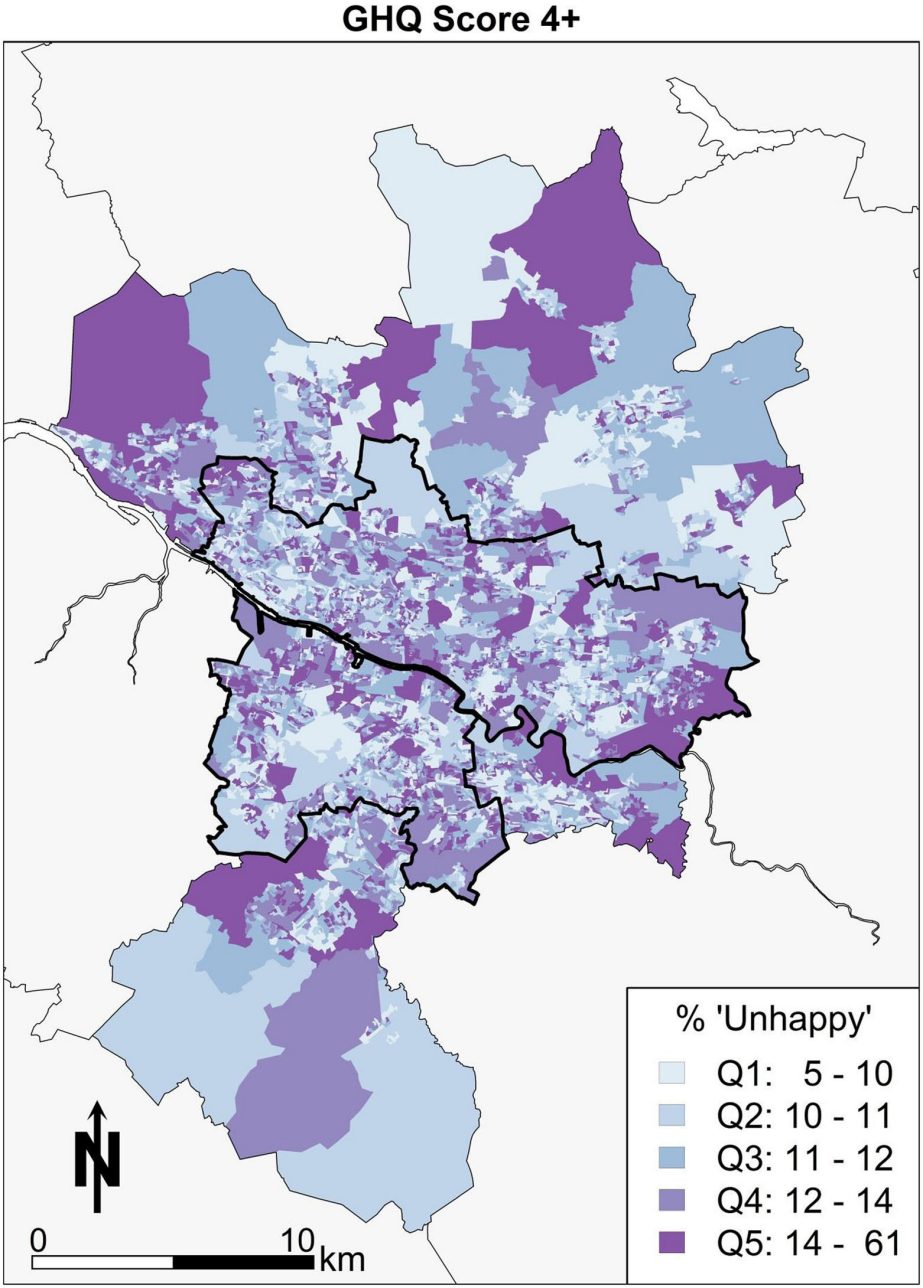
**GHQ score 4 or more (“unhappy”)**.

General health questionnaire (GHQ) scores are a measure of subjective well-being based on a series of questions resulting in a single number summary of mental health, where a higher score denotes increased mental distress. First, the simulated spatial pattern of subjective well-being is visualized as shown in Figure 2. There is a notable series of clusters in the east end of Glasgow. The areas with the lower percentages of individuals (lighter colors) appear to be spread around the west end and to the northern edges of Glasgow, which is what is likely to be expected *a priori* from the socioeconomic geography of Glasgow. In other words, the most deprived areas have worse mental health outcomes. Elsewhere, the pattern of mental well-being appears sporadic in Glasgow with smaller scattered clusters toward Drumchapel for example.

**Figure 3:**
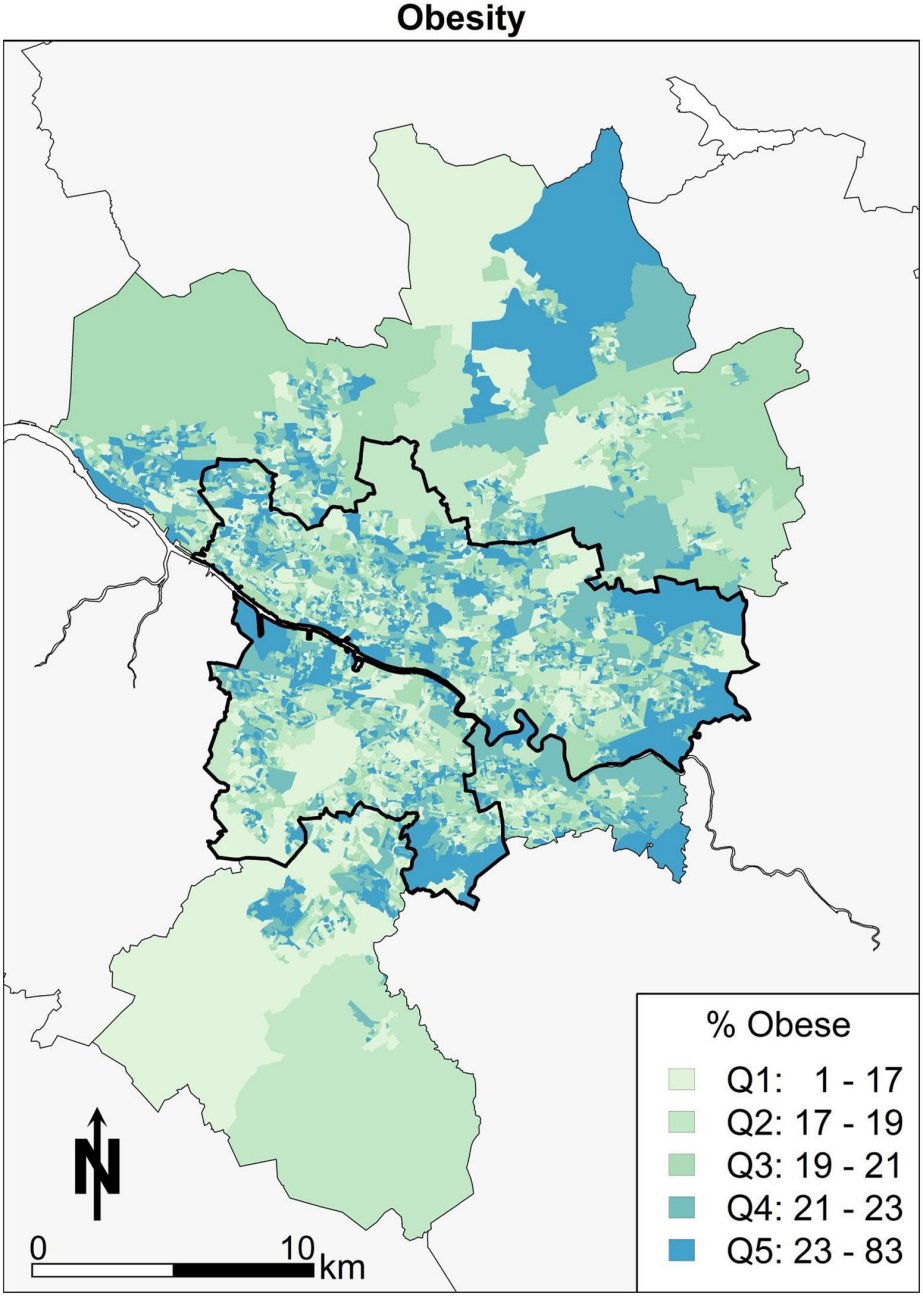
**Obesity**.

Second, the geography of Glasgow in terms of BMI is looked at briefly in this paragraph. Those areas colored darkest (Q5) with large numbers of obese people are in the east of Glasgow in Figure 3, Easterhouse, and Shettleston. Areas with higher proportions of obesity are also concentrated in the Castlemilk area of Glasgow to the south east. There are similar small enclaves of areas in the areas bordering the river Clyde to the western edge on the south side of Glasgow city. The pattern would appear to follow an explanation of poor socioeconomic conditions correlating with obesity in the Glasgow area.

**Figure 4:**
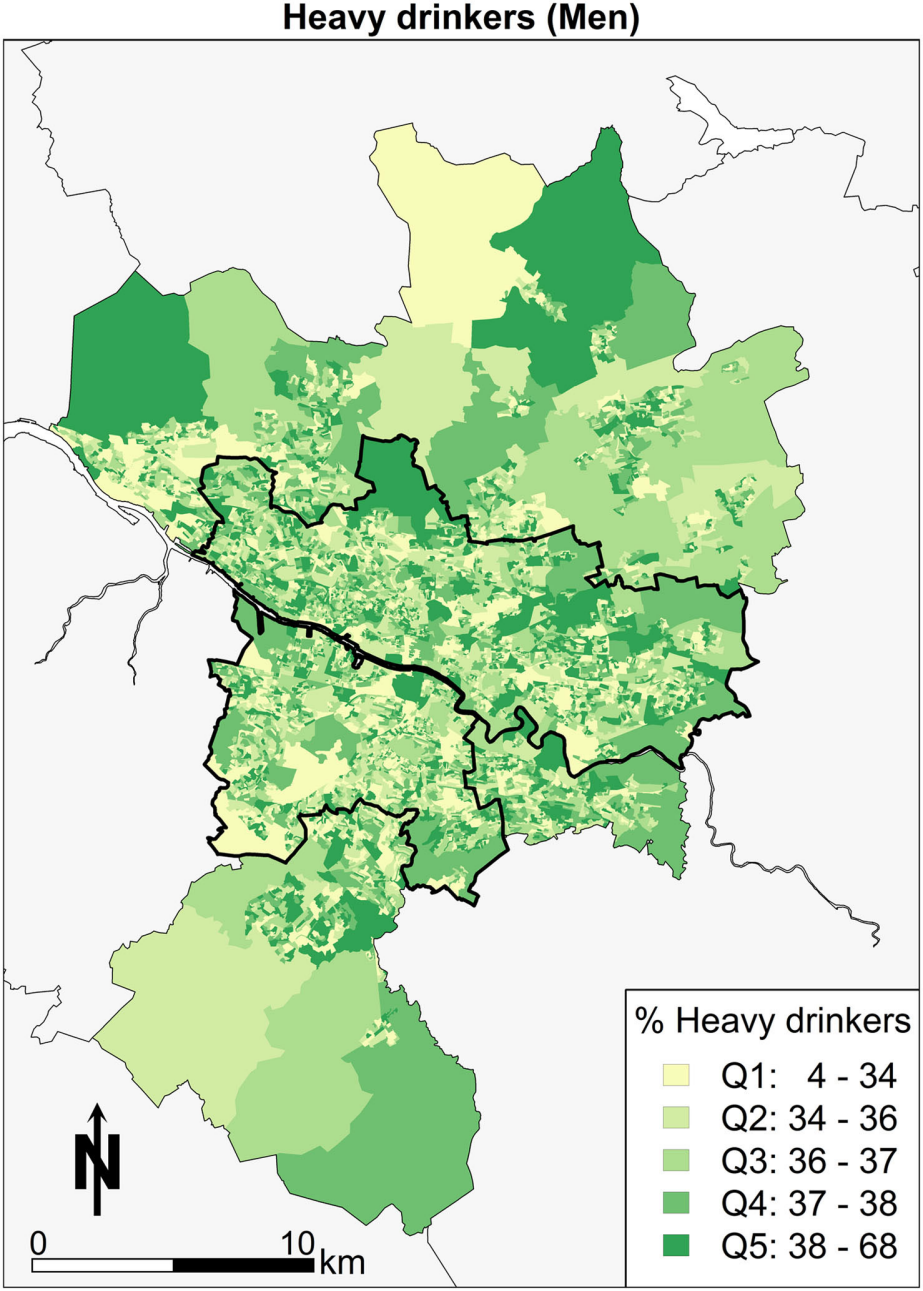
**Alcohol consumption over daily limit (men)**.

Third, the focus moves to the spatial patterns of alcohol consumption in Greater Glasgow. Overall, the summary is that there is little in the way of a clear pattern (Figure 4). The pattern of east end doing “poorly” is not as apparent for this variable. The message overall is that there are few “pockets” of problem drinking, so it is more difficult to conclude that this is linked to the area.

**Figure 5:**
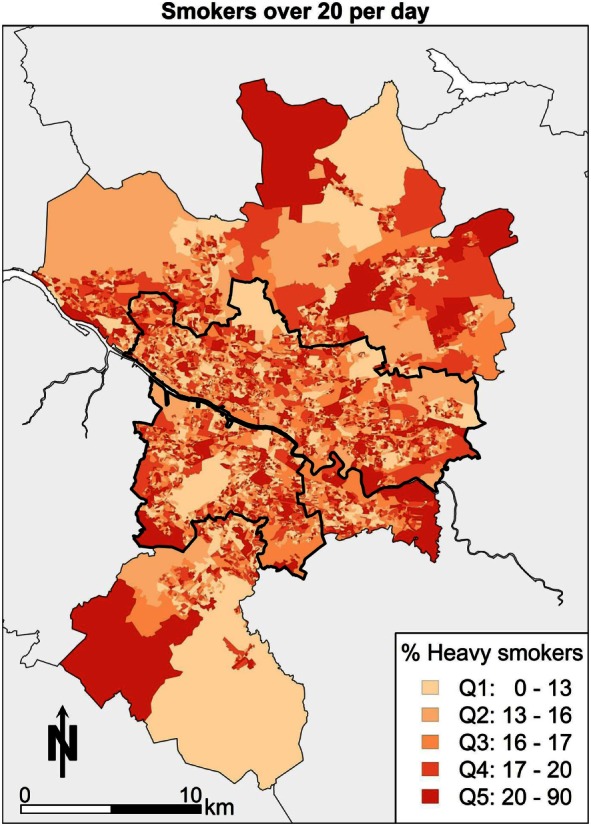
**Heavy smokers**.

Fourth, the geography of smoking in Glasgow in Figure 5 shows smokers using over 20 cigarettes a day. Focusing on the spatial pattern, areas toward Castlemilk in the south east, the east end around Easterhouse, and the parts of the central areas bordering the river Clyde have the highest proportions of heavy smokers.

The spatial patterns demonstrated in each of the estimated health outcomes and behaviors, to a greater or lesser extent, mimic the aforementioned patterns of deprivation. The particular social geography within the Greater Glasgow area is therefore important context to the estimates produced here.

## A Stylised Policy Scenario: Identifying Areas of High Need

**Figure 6:**
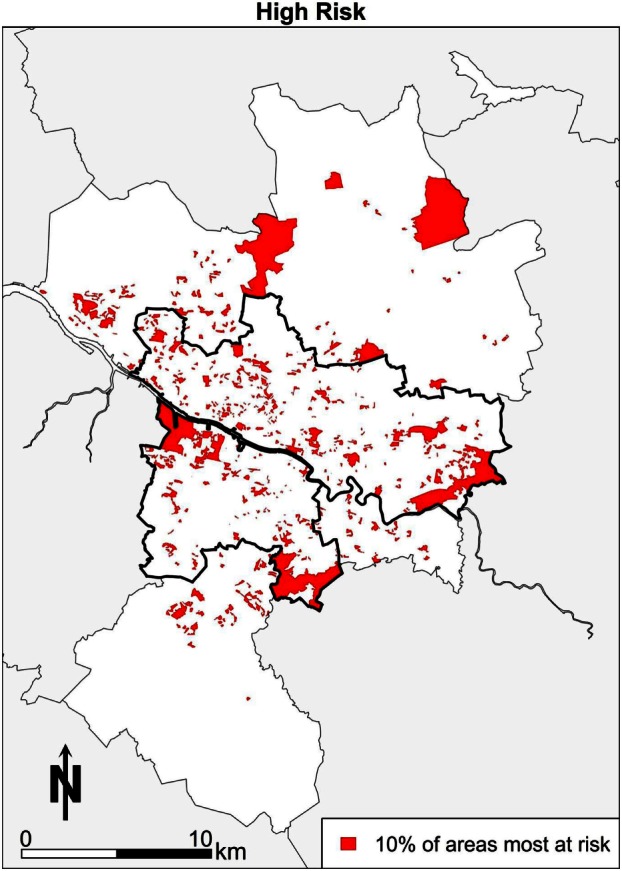
**Combined high risk map**.

This section explores the power of spatial microsimulation in more depth by again demonstrating some of the consideration advantages over more “traditional approaches.” Imagine a policy scenario where the aim is to identify the areas with the most “unhealthy” persons, and the areas in which they reside. This can be achieved in spatial microsimulation modeling. Data can be combined, such that the people who are smoking 20 or more cigarettes a day, drinking more alcohol than the guidelines suggest, have low subjective well-being and also obese simultaneously are selected, then mapped. This combination of factors could be considered “unhealthy,” so finding the areas in which these people live may be a priority so that health policy can target concentrations of “poor” health outcomes. The map in Figure 6 shows the “high risk” areas in terms of health for Greater Glasgow. The spatial pattern in Glasgow shows that some areas stand out visually. There are areas of clustering in places that are expected to feature in the “poor” health end of the distribution, such as areas in the east end of Glasgow, around Easterhouse, and Castlemilk. Other areas, such as Drumchapel, have pockets of “high risk” health features. On balance, the pattern is concentrated more within the city boundary than outside it, punctuated by smaller clusters spread across the city with notable “gaps” (i.e., white space) in the more affluent areas of the city, such as the west end. The pattern does show elements of the other health maps, which is to be expected as it is a combination of all four of the previous health maps of Glasgow. The concentration of “high risk” areas could have important health implications and additional effects on health that smaller isolated clusters may not exhibit would have a much greater effect where there are combinations of “high risk” health. In other words, the combination of high alcohol consumption, smoking, obesity, and poor mental health may well have longer-term effects as well as compounding effects on individual and area-level health. It could be argued that area-based policies, i.e., targeting a specific neighborhood, would work by targeting these “high risk” areas, and this may well have an impact at the national or city level in terms of an improvement to health outcomes more generally.

**Figure 7:**
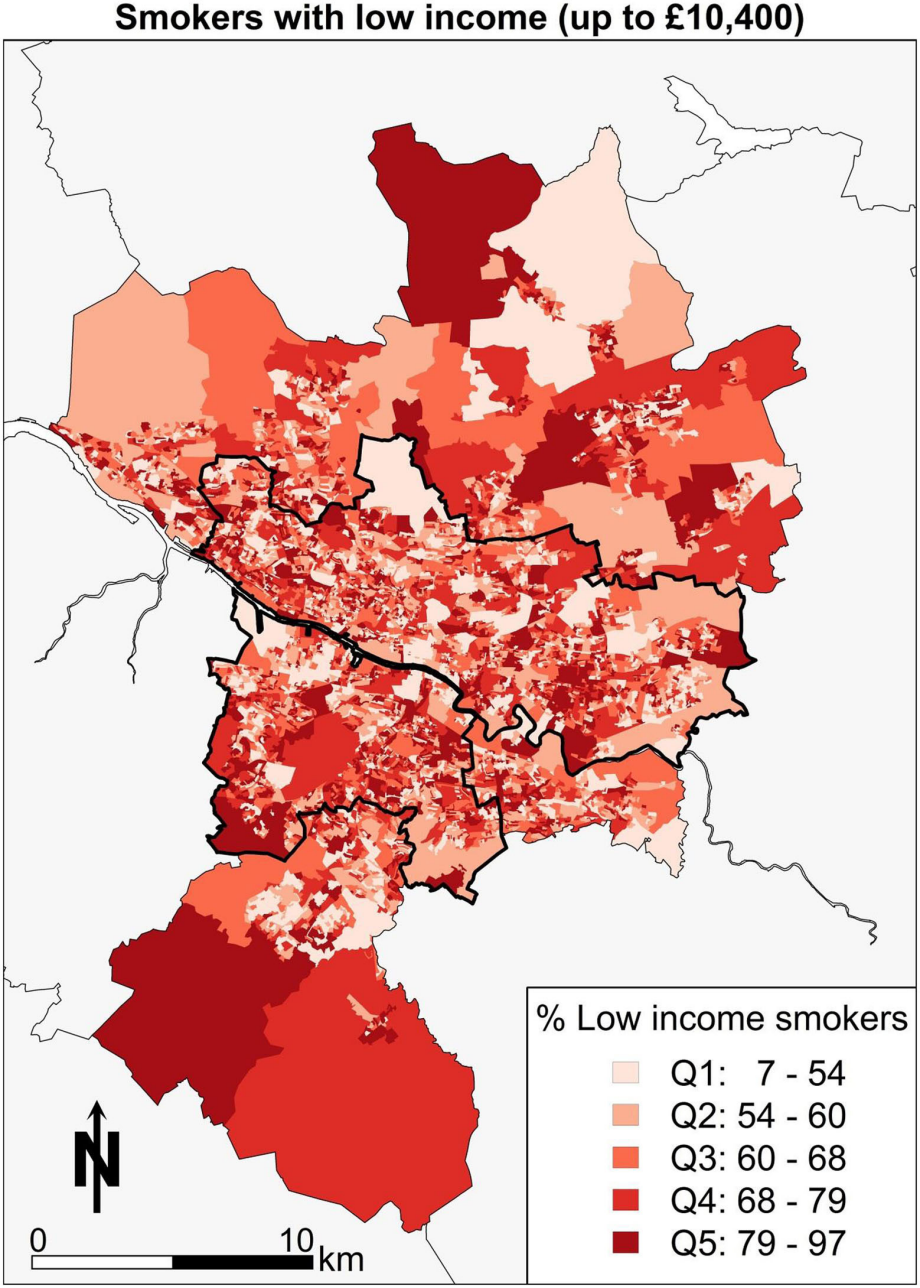
**Low income and smoking**.

A further example of the power of spatial microsimulation is to combine and cross tabulate socioeconomic and health variables geographically. In Figure 7, the map shows the areas with the highest proportions of people who have low income and are smokers. What the map shows is those areas with the darkest reds (Q5) contain between 78 and 96% of people in that category as a proportion of all people in each area. In other words, almost all of the people in some areas of Glasgow are low-income smokers. There is an advantage to know which of those areas are worth focusing resources in terms of stopping smoking services. Areas to the south, such as Shettleston and areas to the East, such as Easterhouse, are highlighted with respect to smoking behaviors and low income.

## Discussion

In 2006, Scotland introduced a nationwide ban on smoking in public places and plans to end tobacco displays in shops as well as to ban sales from vending machines. Scottish studies (41) report that reductions in exposure to secondhand smoke of the order observed in Scotland may generate immediate health gains in the Scottish population as well as longer-term reductions in morbidity and mortality related to secondhand smoke due to the smoking ban. Haw and Gruer (41) argue that quitting smoking is probably the most effective way of reducing secondhand smoke exposure in the home; and that smoking cessation services must continue to be promoted. Additional evidence (30) again supports the thesis that smoke-free legislation has been a success. An option would be to model smokers to better target this group of the population if desired. The use of microsimulation to model smoking rates is not new, as the geography of smoking in Leeds (4) has previously been estimated. The microsimulation of smoking rates in *SimAlba* builds on this type of work and brings it to a Scottish context, which does not appear to have been modeled before. There are also arguments about broader macroeconomic forces, such as income inequality (42), being the cause of a plethora of health and social ills. The debates around greater income inequality leading to higher rates of not just smoking but also poorer mental health outcomes and higher rates of obesity are well rehearsed in the literature.

Another aspect of health that is relevant in Scotland is mental health outcomes. Scotland has high rates of suicide (43) compared to England and Wales. Spatial microsimulation could be used to specifically target “at risk” groups, geographically. Previous modeling has been completed in England (44) showing the spatial patterning of small-area prevalence of psychological distress and alcohol consumption. Also, there have been attempts to estimate happiness in Scotland with the use of spatial microsimulation (34) by combing the British Household Panel Survey (BHPS) with census data. What the analysis in this paper adds is a more complete picture of other health variables, also using a health-specific survey data set (SHS instead of the BHPS), and building on the existing work from elsewhere in the UK.

Alcohol policy is also of particular policy relevance due to the debates on the introduction of a minimum price per unit of alcohol (45). The Scottish government previously introduced an alcohol bill to try and begin the process of legislating for the changes needed, such as the minimum price per unit of alcohol. In the background of alcohol consumption debates is the framework of the recommended daily limits for alcohol consumption of no more than 3 or 4 U (2 or 3 U) of alcohol per day for men or women, respectively. The analysis presented here shows the estimated geographic location and the characteristics of people who drink over the guideline limits adding extra depth to the existing data. As noted by Katikireddi and McLean (28), there is a lack of empirical evidence in this regard which, it could be argued, can be addressed by spatial microsimulation models (e.g., *SimAlba*).

Obesity is a growing problem worldwide. It is also a costly problem with between 0.7 and 2.8% of a country’s total health-care expenditures being spent on this health issue (46). There are complex pathways and dynamics behind the determinants of obesity (47) that explain the doubling of the rate, since 1980 worldwide, to a rate of around 20% in most developed economies, such as the context explored here (48). More concerning is that patterns among children and adolescents continue to show growth in rates of obesity (49). Interestingly, when looking at the relationship between play areas and deprivation and subsequent links to childhood obesity (50), it was found that more deprived areas are better provided for, but, the quality has not been accounted for, neither has the lack of private green space relative to more affluent areas, so causal pathways in some instances are unclear. Moreover, in Glasgow, there is evidence to suggest that more deprived neighborhoods are no more likely to be exposed to energy dense out-of-home eating outlets (51). So, simple explanations relating to providing more play areas and reducing exposure to out-of-home eating outlets are not sufficient explanation for increasing obesity rates, The *SimAlba* model adds to a literature on simulated obesity rates for small areas seen elsewhere in the UK (38). More recent literature (52) has continued in a similar vein, emphasizing the importance of designing policies targeted at the small area level, but also that account for population group differences simultaneously.

## Conclusion

A comprehensive dataset, such as that generated by *SimAlba* that provides data on health-related behaviors for individuals and small areas in Scotland, has previously not been available. Although the data simulated are now updated, it provides an important addition to understanding the health behaviors at small area geographies. The missing piece of the puzzle has always been that reliable small area data on all these types of behaviors and conditions are not collected, except, for very broadly, by the Census, which exists for self-reported health for example. What spatial microsimulation adds is the lower level, small area geography, the ability to examine both composition, and context simultaneously.

**Figure 8:**
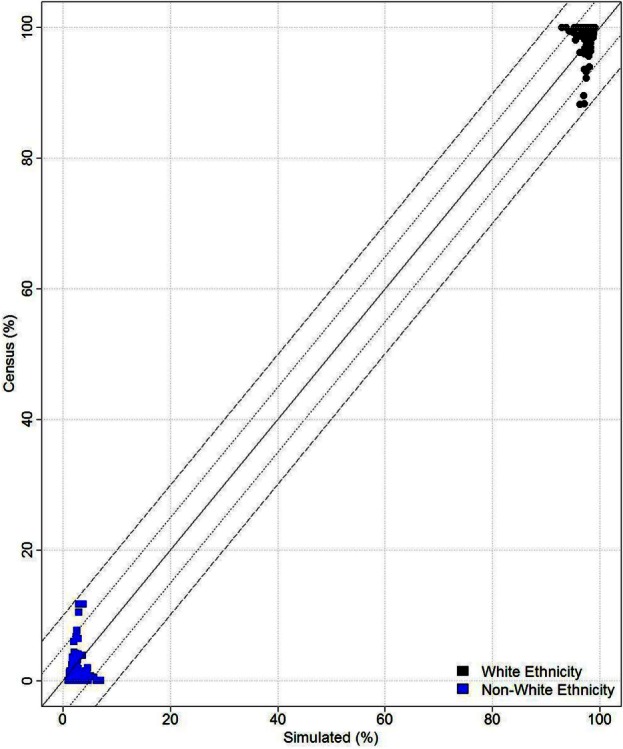
**Validation results**.

Nevertheless, it should be noted that one concern with spatial microsimulation is the issue of validation – how accurate simulated data are – and how to assess quality of outputs. This concern has been addressed or discussed in papers looking at deterministic reweighting models (6), and there are ongoing debates (53) on this specific issue. Therefore, the main limitation of microsimulation is that it is difficult to verify that the outputs against what the real population data may be. The paradox of this approach is that the reason the data are simulated in the first instance is that it is difficult or too expensive to collect. On balance, the *SimAlba* model appears to produce reasonably accurate microsimulated data where validation or use of a proxy variable to test results have been possible as demonstrated elsewhere (35, 36), as well as seen in Figure 8.

The analysis presented provides policy makers with an indication of those areas where individuals with a variety of health outcomes (smoking, alcohol consumption, obesity, and mental well-being) are potentially living within Glasgow, and this information could potentially be used to target smaller area interventions compared to a universal intervention. Subjective well-being (measured by GHQ 12 score) has also been examined, and there does not appear to be any other study in which estimated GHQ scores at such small areas in Scotland. Alcohol consumption was also modeled using the *SimAlba* framework. The simulation of data of this nature could be considered valuable to policy makers in showing the differing spatial concentrations of problem drinkers. Furthermore, obesity and various weight categories were simulated using *SimAlba*. The analysis provides an original dataset to explore health outcomes and behaviors in Scotland at either the individual-level or small area-level geography. The estimation of health-related variables; smoking, alcohol, happiness, and obesity at small area level geography is a step forward in understanding what the patterns of health behaviors or health indicators are likely to be. There is still significant potential to use the microdataset created for future research in a variety of fields. The SimAlba model is also able to estimate other variables, which are present in the SHS (e.g., regular exercise), but this would require a modified spatial microsimulation model. The model presented here could also be used as a basis for future modeling work or as the basis of a framework for other survey data sources, for example, to look at spatial and social patterns of tobacco cessation, condom use for disease prevention, seat belt use, or breastfeeding.

## Author Contributions

MC collected and analyzed data and wrote the first draft; DB made suggestions regarding the analysis and interpretation and also co-authored and edited the manuscript.

## Conflict of Interest Statement

The authors declare that the research was conducted in the absence of any commercial or financial relationships that could be construed as a potential conflict of interest.

## Footnotes

^1^The model is named *SimAlba* as Alba is the Scots Gaelic name for Scotland, and it is a Spatial Microsimulation model of Scotland.
