# Corrigendum: SimAlba: A Spatial Microsimulation Approach to the Analysis of Health Inequalities

**DOI:** 10.3389/fpubh.2017.00340

**Published:** 2017-12-22

**Authors:** Malcolm Campbell, Dimitris Ballas

**Affiliations:** ^1^GeoHealth Laboratory, Department of Geography, University of Canterbury, Christchurch, New Zealand; ^2^Department of Economic Geography, Faculty of Spatial Sciences, University of Groningen, Groningen, Netherlands

**Keywords:** spatial microsimulation, urban health inequalities, health policy, scotland, geographic information systems, small area microdata

In the original article, we neglected to include the Acknowledgments section.

## Conflict of Interest Statement

The authors declare that the research was conducted in the absence of any commercial or financial relationships that could be construed as a potential conflict of interest.

